# The Devil in the Details: Public Health and Depression

**DOI:** 10.3389/fpubh.2014.00192

**Published:** 2014-10-14

**Authors:** Andreas Vilhelmsson

**Affiliations:** ^1^Department of Clinical Sciences, Division of Social Medicine and Global Health, Faculty of Medicine, Lund University, Malmö, Sweden

**Keywords:** depression, medicalization, mental disorders, overdiagnosis, overtreatment, public health

## Background

Mental disorders have been increasingly portrayed by the World Health Organization (WHO) and health researchers as a growing burden to global public health (1–5). Just the sheer economic impact of mental disorders is significant; it is expected to cost almost a third of the projected US$47 trillion incurred by all non-communicable diseases by 2030 (6). American and European research indicated in 2005 that 26–27% of the adult population suffers from a diagnosable mental disorder, representing over 57 million Americans and almost 83 million Europeans (7, 8). The European research was later revised in 2011 to 38% (approximately 160 million Europeans) by including mental diagnoses usually not analyzed in these kinds of studies, such as insomnia and alcoholism (9). In Sweden as well as other countries, milder mental symptoms are now being frequently reported as common occurrences (10, 11), especially among youth and the elderly (12, 13). These milder symptoms are increasingly becoming highlighted as important; research (for example, Swedish and American) have suggested that early mental ill health can predict more severe mental illness and mental disorders (such as major depression) later in life (14–16) and even premature death (17). It is, therefore, often argued that early signs of mental ill health need to be acknowledged and treated to prevent the onset of mental disorders (14–16, 18–21).

Depression is the most common of the affective disorders, which are defined as disorders of mood rather than disturbances of thought or cognition (22). Depression is estimated to have a point prevalence of about 5% in the general population, and a lifetime risk of about 15% (23). Globally, more than 350 million people of all ages are believed to suffer from depression (24). Just in the European Union, a yearly prevalence of 6.9% of depression is estimated to affect 30.3 million inhabitants (9).

Overall, the WHO now ranks depression as one of the most burdensome diseases in the world, and the organization has for some time projected and warned that depression is predicted to be the highest-ranking disease problem in the developed world by 2020 (1, 2).

This description is, however, not without controversy, and some scholars are skeptical of how, for instance, depression is viewed as an increasing widespread ill health problem (25–29). The aim of this article is to discuss some of these issues.

## Discussion

### The anatomy of public health

As Dubos argues in his classic book *Mirage of Health*, the myths of Hygeia and Asclepius symbolize the never-ending oscillation between two different points of view in medicine: health as the natural order of things and health as something to be restored by correcting an imperfection (30). The modern followers of Hygeia can be understood as practitioners of public health and the medical professionals as followers of Asclepius (31). It is sometimes argued that with the exception of the specialties of public health and family medicine, the focus of modern medicine is mainly on the individual patient, rather than relating their situation to their families, communities, or the wider society (32). However, while public health medicine has long engaged in strategies of disease prevention and health promotion, more individualized practices of risk are argued to have become a central dimension of the politics of life in the twenty-first century (33). Increasingly, we have come to regard simply being at risk of future disease as being a disease in its own right (28). Diagnostic labels now go beyond disease itself to include risk factors for disease, sometimes giving rise to a new source of social identity, namely a pre-disease (34). This is what critics argue is underway with the introduction of preconditions for major depression in DSM-5 (35). Focusing on preconditions for disease may further increase what the German sociologist, Ulrich Beck, has called the “risk society” (36) and in a global approach “world risk society” (37); a society structured through individualization where a social crisis appears as an individual crises, no longer perceived in terms of their rootedness in the social realm. Thinking of depression in terms of risk is related to the problematization of depressive illness in the population and as a public health issue (38). But by trying to assess potential risk factors for disease and disorders at earlier stages, the concepts of illness and risk may become increasingly blurred (39).

### The ghost in the machine

A medical diagnosis is perhaps most readily recognized as the official label that classifies disease as a medically related problem, and is the foundation from which sense-making and experiences are crafted (34). A diagnosis can validate a patient’s perception of her symptoms by giving her experience a name, and equally, it can pathologize routine lived experience, such as fluctuations in one’s mood (40). Medical encounters usually take place within a system where diagnostic handbooks and short form tests are used as a fast way of judging a person’s health status, a system that allows and encourages doctors to swiftly choose a diagnosis without a comprehensive investigation of the whole situation surrounding the patient. As argued, for instance, by psychiatrist David Healy, guidelines and protocols are now part of an “industrialization of health care” as he calls it (41). This is not the purpose for which these handbooks were intended. The DSM was issued as a manual for guiding decisions regarding diagnosis, but has more often been used as a steering document for diagnosis. For instance, it is stated in the DSM-IV that, “*It is important that DSM-IV not to be applied mechanically* … *and are not to be used in a cookbook fashion*” (p. xxxii) (42). By using the DSM as a “fast track” to diagnosis, one may end up with a problem with not just overdiagnosis, but also overtreatment. In the medical encounter, the doctor may judge it to be more dangerous not to treat someone who may prove to be ill than to treat them when actually there is no need to do so, and as a precaution and in fear of relapse recommend long-term use of medicines (43). Research has suggested that the act of prescribing in itself might also suggest a biological basis for a problem (44), and that it appears that doctors are less willing to consider non-drug treatments if drug therapy is available, even when there is no evidence that pharmacotherapy is superior (45).

### Risk of overdiagnosis and overtreatment

One pathway to overdiagnosis can be through disease boundaries being widened and treatment thresholds lowered to a point where a medical label and subsequent therapy may cause people more harm than good (28). This broadening of diagnostic criteria is argued to reflect medicalization as much as discovery of previously undetected sick people (35, 46–49). Non-medical problems have become medical ones with risking leading to overdiagnosis and overtreatment as the definition of what constitutes an abnormality gets increasingly broader (50).

Concern for the harm and costs of overdiagnosis and overtreatment is now gaining momentum, as the discussion of risk assessment and suggestions of pre-disease progress in the scientific debate (51, 52). Missed, delayed, or incorrect diagnoses can lead to inappropriate patient care, poor patient outcomes, and increased costs (53). In the United States, it has been estimated that between $158 billion and $226 billion was wasted on overtreatment in 2011 (54), and that the cost of medicalization in 2005 corresponded to almost 4% of the total domestic expenditures on health care that year, or $77 billion (55). Thus, overdiagnosing depression “just in case” or because of a risk assessment may take its toll both health-wise and financially. One study found overdiagnosis and overtreatment of depression to be common in community settings in the U.S. (56).

Whether the public benefits from taking more and more medicines for increasingly broadly defined disease is open to serious question. Critics like Marcia Angell argues, for instance, that one could make a strong argument that Americans with minor ailments suffer more from overmedication, and all the side effects and drug interactions that go with it, than from undermedication (48). Thus, overdiagnosing depression “just in case” or because of a risk assessment may take its toll both health-wise and financially.

### The devil in the details

Maybe, the devil is in the details. The “one in four” figure for mental illness prevalence, widely quoted as it is, has an unclear origin (57). Is it even reasonable that 27% of the American and European population is estimated to suffer from mental disorders (or 38% Europeans depending on how many disorders are included)? Or is that approximately 8.5% of the Nordic population prescribed as antidepressant medication? (58). Or does it instead tell us something about the contemporary global community and our view of health and ill health? Pharmaceutical companies have for some time been accused of “disease mongering” (28, 59), whereby a “new condition” is promoted as a major public health problem in order to create a market for treatment, often without the public’s knowledge, sometimes referred to as the “public healthification” of social problems (60). And there is also the issue of alleged financial ties not only between the members of the DSM panels and the pharmaceutical industry (61, 62) but also between industry and doctors responsible for clinical practical guidelines for other medical conditions as well (63).

If normal events are misdiagnosed as depression, this will risk leaving those who are depressed untreated (extended waiting lists to health care, wrong medications, or lack of resources) and thereby create undertreatment and overtreatment simultaneously. If depression is going to be viewed as a growing public health problem, there needs to be a distinction between ill health problems that are medical problems and those that are not.

## Summary

It is evident that there are conflicting views regarding the officially proclaimed widespread existence of mental disorders, and depression in particular. On the one hand, we face descriptions of a growing public health burden and risk, but on the other hand we have descriptions of overdiagnosis and overtreatment. Certainly, from a public health perspective anyway, a medical approach to mental disorders would be troublesome, since increasing medicalization furthermore risks individualizing mental problems that may have other sources and thereby moves the focus away from the social and political context of ill health, for instance, poverty and inequality. For the sake of public health, arguments for increased diagnosis must therefore be related to a possible danger of medicalizing social problems and life crises. By including people with mild problems in estimates of mental illness, we risk losing support for treating those people who have legitimate disorders.

## Conflict of Interest Statement

The author declares that no support was given from any organization for the submitted work; that there are no financial relationships with any organizations that might have an interest in the submitted work; that no other relationships or activities exist that could have influenced the submitted work.
